# Usability Issues in Evidence-Based Psychosocial Interventions and Implementation Strategies: Cross-project Analysis

**DOI:** 10.2196/37585

**Published:** 2022-06-14

**Authors:** Sean A Munson, Emily C Friedman, Katie Osterhage, Ryan Allred, Michael D Pullmann, Patricia A Areán, Aaron R Lyon, UW ALACRITY Center Researchers

**Affiliations:** 1 Department of Human Centered Design & Engineering University of Washington Seattle, WA United States; 2 Advanced Laboratories for Accelerating the Reach and Impact of Treatments for Youth and Adults with Mental Illness (ALACRITY) Center University of Washington Seattle, WA United States; 3 Department of Psychiatry and Behavioral Sciences University of Washington Seattle, WA United States; 4 Department of Family Medicine University of Washington Seattle, WA United States; 5 See Acknowledgments

**Keywords:** evidence-based psychosocial interventions, usability, implementation strategies, mental health, human-centered design, implementation science

## Abstract

**Background:**

People often prefer evidence-based psychosocial interventions (EBPIs) for mental health care; however, these interventions frequently remain unavailable to people in nonspecialty or integrated settings, such as primary care and schools. Previous research has suggested that usability, a concept from human-centered design, could support an understanding of the barriers to and facilitators of the successful adoption of EBPIs and support the redesign of EBPIs and implementation strategies.

**Objective:**

This study aimed to identify and categorize usability issues in EBPIs and their implementation strategies.

**Methods:**

We adapted a usability issue analysis and reporting format from a human-centered design. A total of 13 projects supported by the National Institute of Mental Health—funded Accelerating the Reach and Impact of Treatments for Youth and Adults with Mental Illness Center at the University of Washington used this format to describe usability issues for EBPIs and implementation strategies with which they were working. Center researchers used iterative affinity diagramming and coding processes to identify usability issue categories. On the basis of these categories and the underlying issues, we propose heuristics for the design or redesign of EBPIs and implementation strategies.

**Results:**

The 13 projects reported a total of 90 usability issues, which we categorized into 12 categories, including complex and/or cognitively overwhelming, required time exceeding available time, incompatibility with interventionist preference or practice, incompatibility with existing workflow, insufficient customization to clients/recipients, intervention buy-in (value), interventionist buy-in (trust), overreliance on technology, requires unavailable infrastructure, inadequate scaffolding for client/recipient, inadequate training and scaffolding for interventionists, and lack of support for necessary communication. These issues range from minor inconveniences that affect a few interventionists or recipients to severe issues that prevent all interventionists or recipients in a setting from completing part or all of the intervention. We propose 12 corresponding heuristics to guide EBPIs and implementation strategy designers in preventing and addressing these usability issues.

**Conclusions:**

Usability issues were prevalent in the studied EBPIs and implementation strategies. We recommend using the lens of usability evaluation to understand and address barriers to the effective use and reach of EBPIs and implementation strategies.

**International Registered Report Identifier (IRRID):**

RR2-10.2196/14990

## Introduction

### Background

Many people seeking care for mental health problems prefer psychosocial interventions [1-5]. Evidence-based psychosocial interventions (EBPIs) have been shown to be effective in numerous studies; however, despite these preferences and evidence supporting their use, EBPIs remain unavailable to people in most service settings, especially nonspecialty or integrated settings, such as primary care [6] and schools [7,8], where most mental health care is delivered [9].

Non–mental health settings differ from mental health service contexts for which EBPIs are typically developed. The differences in service settings often contribute to a poor contextual fit, low rates of adoption, and sustained use [10,11]. Furthermore, most EBPIs are complex interventions that require ongoing support to ensure their quality. Clinicians face difficulties in learning and adopting these new practices, which further limits EBPI availability [12]. Despite decades of research intended to address these and other barriers, the many implementation strategies that target these barriers are often cumbersome and costly processes to deliver [13,14], resulting in few cost-effective strategies [15] and a lingering science-to-service gap.

Within the University of Washington (UW) Advanced Laboratory for Accelerating the Reach and Impact of Treatments for Youth and Adults with Mental Illness (ALACRITY) Center [16], we believe that new approaches are required to address these barriers. We set out to apply a core concept from human-centered design (HCD) to understand and improve EBPIs: *usability*. Usability is the degree to which a program can be used easily, efficiently, and with satisfaction/low user burden by a particular end user [17]. The concept of usability and techniques for assessing the usability of a system and then ideating, designing, implementing, and evaluating usability fixes has been central to the widespread success of modern digital tools, and we argue that usability metrics and assessment methods are broadly relevant.

Drawing on the results from 13 projects in the Center, we identified 13 categories of usability issues in EBPIs and their implementation strategies. We propose heuristics to prevent or mitigate such issues in the future design of EBPIs and their implementation strategies. We also present our development and use of the usability issue format as a resource for researchers.

### Overview of EBPIs

EBPIs are complex health interventions involving interpersonal or informational activities and techniques [12]. In mental and behavioral health, EBPIs target biological, behavioral, cognitive, emotional, interpersonal, social, or environmental factors to reduce the symptoms of these disorders and improve functioning or well-being. Examples of EBPIs to address mental health concerns include parent training protocols to address children’s disruptive behavior problems [18] and cognitive behavioral therapies for adults with anxiety or depression [19].

Over the past few decades, hundreds of EBPIs have been developed for use with youth and adults; however, their adoption, high-fidelity delivery, and sustained use in routine service delivery remain low [20-22]. This is because of a variety of multilevel barriers and facilitators (ie, determinants), ranging from the characteristics of the interventions themselves to broader social, political, and policy influences [23]. Recognizing these struggles, researchers and practitioners have increasingly worked to develop *implementation strategies* such as methods or techniques used to enhance the adoption, implementation, and sustainment of a clinical program or practice by addressing determinants [14,24]. Implementation strategies are complex interventions that are largely socially mediated and interpersonal or informational [14,25]. More than 70 unique implementation strategies have been developed for use across a variety of health care delivery settings [26,27]. Examples of strategies include informational meetings or training, audits, and feedback processes.

The consistent and effective use of both EBPIs and implementation strategies has been suboptimal [18,28], and additional approaches are needed to improve practitioner and service recipient engagement with these innovations. Both EBPIs and strategies have a common disconnect between the settings in which they were developed, which are most often academic medical centers or other research settings, and the real-world contexts in which they may ultimately be deployed [29-31]. Few contemporary approaches exist that highlight and address the design quality of both EBPIs and implementation strategies [16].

### HCD and Usability

HCD (or the closely related field of user-centered design) is a field that has produced methods of developing compelling, intuitive, easily adopted, and engaging products, services, and tools [32]. HCD has appropriated, adapted, and developed methods for systematically understanding users and other interested or affected parties and contexts, ideating and designing innovations to address needs, opportunities, and problems, and then evaluating the resulting innovations. As a field, HCD excels in improving innovation-user and innovation-context fit.

Usability—the extent to which a system or service can be used by specific people to achieve specified goals with effectiveness, efficiency, and satisfaction within a specified context of use (ISO 9241-11:2018) [17]—is a core concept at all stages of the HCD process. Understanding what makes existing solutions unusable for some people, or what would make an experience more usable, can drive problem identification and innovation. As innovations move forward in the design process, they must be evaluated for usability at each stage, or the resulting innovation may have limited adoption or harmful or even fatal errors.

Several approaches have been developed to ensure the usability of designed systems. This includes usability evaluation (or usability testing), in which users interact with prototypes or implemented systems in scenarios or in open-ended use. This use could occur in the laboratory or field. Evaluations often begin before target users are directly engaged. Designers are trained in common usability issues and heuristics, or guidelines, that help prevent these issues. Designers and user experience professionals may use these guidelines in the formal heuristic evaluation of prototypes by walking through parts of the interface to identify and address potential issues. Outside formal heuristic evaluation, most designers have internalized common heuristics [33], which helps pre-empt many potential usability issues as they work. In cognitive walk-throughs [34], which is another form of early-stage evaluation, domain experts walked through the proposed interface, often guided by specific scenarios, to identify potential usability issues.

Although these techniques were developed primarily in the design and evaluation of digital systems (or hybrid digital-physical systems), including in mental health [35], they are also promising for understanding what facilitates and inhibits the successful implementation, adoption, and use of EBPIs. Recent studies have applied HCD to EBPIs to enhance usability, decrease burden, and increase contextual appropriateness [1,13,25]. This research has also shown how HCD evaluation approaches, such as cognitive walk-throughs, can be adapted to identify usability barriers in implementation strategies [25].

In this study, we sought to apply usability evaluation methods to identify usability issues across a range of EBPIs and their implementations. We propose that this research can help to understand the ways in which EBPI design and implementation create barriers to their use by patients, clinicians, and other primary and secondary users and that such an understanding can led to the development of heuristics that improve future EBPI design and implementation.

Specifically, we asked the following: (1) what are the common usability issues in EBPIs and implementation strategies for mental and behavioral health, and (2) what are the characteristics of the highest-severity usability issues?

In this paper, we present the results and reflections of our investigation on the development and use of a usability issue–reporting format for mental and behavioral health interventions.

## Methods

### Overview

Drawing from 13 projects examining the usability of EBPIs, such as behavioral activation (BA) and problem-solving therapy (PST), and their implementation strategies, project teams and Center researchers identified 90 usability issues. We clustered these into 13 categories of issues. From these clusters, we developed heuristics that we propose can be used by EBPI designers and implementers to guide the design, redesign, and implementation of EBPIs across a range of accessible community settings.

### Discover, Design/Build, and Test Process and Data Sources

#### Overview

The UW ALACRITY Center organized the work in these projects according to the Discover, Design/Build, and Test (DDBT) model, which draws from both implementation science (IS) and HCD [16]. The DDBT model is a phased approach to intervention and strategy redesign that uses methods from HCD and is informed by the Consolidated Framework for Implementation Research model, including the Consolidated Framework for Implementation Research intervention, individual, inner setting, and process constructs [23]. In the Discover phase, redesign teams, comprising EBPI experts and HCD researchers, worked to identify the usability challenges they experienced when interacting with the EBPI. Strategies for usability identification included but were not limited to focus groups, in situ use of the EBPI, cognitive walk-throughs, interviews, and contextual observations. Solutions for the usability challenges were identified in the Design/Build phase, whereby redesign teams worked together to create initial prototype solutions that users iteratively used and redesigned.

#### Data Sources

Data were provided by investigators on 13 projects: 2 pilot projects and 11 seed projects funded through an open call at the UW ALACRITY Center. These studies focused on (1) improving the implementation or usability of an existing EBPI and (2) ≥1 key issue of interest to the Center (clinician capacity, usability, and sustained quality). Each project specified a conceptual model with one or more hypothesized mechanisms that improved the clinical or implementation outcomes. All proposals were peer reviewed and assigned a mentor from the ALACRITY Center’s core team of investigators. The projects and processes by which project teams described and reported usability issues are explained in the following sections.

#### DDBT Procedures

Although we standardized the format for describing usability issues, we did not prescribe specific methods for each project team to use in the stages of DDBT. Consequently, teams used a range of methods, including interviews, focus groups, cognitive walk-throughs [34], behavioral rehearsals [36], asynchronous remote communities [37], and various other approaches to understanding the usability of EBPIs and implementation strategies [38]. Different methods are appropriate for different groups (eg, for people with barriers to accessing technology, the asynchronous remote community method, which requires ongoing engagement through technology, would hinder participation, although it was ideal for long-term engagement with a group that regularly uses social media to keep in touch [37]). The Center provided teams with support from Center investigators, mentors, and professional staff for the selection and application of HCD and implementation of scientific methods in their work. We also recommended the use of the Intervention Usability Scale [39], System Usability Scale [40], User Burden Scale [41], Implementation Strategy Usability Scale [25], Acceptability of Intervention Measure, Feasibility of Intervention Measure, and Intervention Appropriateness Measure [42] to assess the usability and implement ability of an intervention.

We also did not prescribe the design *fidelity* of the interventions and implementation strategies to be investigated. Design fidelity (not to be confused with treatment fidelity) refers to the details, completeness, and functionality of a prototype. In design, lower-fidelity prototypes can support exploring several different design options at a lower cost. They are valuable for identifying major incompatibilities between design and user needs. In the projects in our study, low-fidelity prototypes included storyboards, mock-ups of digital and/or paper tools intended to support a treatment approach, or even just textual scenarios describing potential changes. Other teams used mock-ups of digital or other artifacts designed to support the implementation of an EBPI or full interactive prototypes of digital services or training designed to support the implementation of an EBPI. However, other teams reviewed the recordings or transcripts of therapy sessions and interviewed clients using existing therapy in practice.

### Overview of the Projects

The 13 projects focused on EBPIs and implementation strategies (Table 1). Among the studied EBPIs, approximately half examined BA [43-45] (3/13, 23% of projects), PST [46] (2/13, 15% of projects), or another EBPI based on one of these (1/13, 8% of projects). These interventions were accessible in many ways: PST and BA are used in nonspecialty settings such as primary care, and our research partners (eg, Seattle Children’s Hospital) also often use BA.

**Table 1:** Our data set included usability issues reported by 13 University of Washington Accelerating the Reach and Impact of Treatments for Youth and Adults with Mental Illness Center projects (N=90 issues).

Project	Setting	EBPI^a^	Implementation strategy	Methods	Issues reported, n
Task sharing with BA^b^ (R34, Areán and Gonzalez)	Rural primary care clinics	BA	Task sharing: shifting more tasks from therapist to care manager to more efficiently implement BA	Qualitative interviews with therapists and care managers during the “Discover” phase	6
PST^c^ support tool (R34, Bennett, Raue, and Munson)	Primary care clinics	PST	PST aid: a web-based tool designed to support the use of PST	Observations and qualitative interviews with clinicians during the “Discover” phase	6
Designing and evaluating an asynchronous remote communities approach to behavioral activation with clinicians and adolescents at risk for depression (R03, Jenness and Kientz)	A hospital or large urban health system	BA	Asynchronous remote communities: offer peer, automated, and clinician support between sessions	Discover: 2 asynchronous remote community studies [47] Design and build: iterative design, build, and usability evaluation of interactive prototype Test: a pilot study, collecting data on feasibility, usability, user burden, acceptability, and symptom outcomes [48]	3
Using human-centered design for technology-enabled behavioral treatment of depression in urban and rural cancer centers (R03, Hsieh and Bauer)	Urban and rural cancer centers delivering collaborative care	BA	N/A^d^	Discover: interviews with 29 stakeholders across 3 groups Design: parallel journeys framework as a conceptual design framework	11
Discovering the capacity of primary care frontline staff to deliver a low-intensity technology-enhanced intervention to treat Geriatric depression (R03, Renn and Zaslavsky)	Primary care	Mobile motivational physical activity–targeted intervention (based on BA)	Task sharing: implementation using frontline primary care staff such as nurses and medical assistants	Discover: focus groups and interviews with 24 stakeholders Design/build: halted because of the COVID-19 pandemic	2
mHealth^e^ in West Africa: developing an evidence-based psychosocial intervention toolkit (R03, Ben-Zeev and Snyder)	Ghanaian prayer camps	Multiple digitally delivered components of evidence-based interventions for psychosis	N/A	Discover: observations and qualitative interviews with 18 healers [49] Design: co-design sessions with 12 healers Build: prototype Test: usability testing with 12 healers	3
Iterative redesign of a behavioral skills training program for use in educational settings (R03, Bearss and Locke)	Elementary school special education classrooms	The RUBI^f^ protocol	N/A	Discover: demonstration study of RUBI with mixed methods feedback [50] Design: collaborative redesign feedback sessions; demonstration study of revised RUBI in Educational Settings with mixed methods feedback	4
Increasing the usability and cultural relevance of an EBPI for suicidality in schools (R03, Brewer and Jones)	High schools	CAMS^g^	N/A	Discover: contextual observations and qualitative interviews with school-based clinicians and focus groups with high school students [51] Design/build: usability testing of unadapted CAMS SSF^h^ with school-based clinicians, followed by a co-design session with school-based clinicians and usability testing of the adapted CAMS SSF with school-based clinicians	4
Improving the usability of decision support for PTSD^i^ in primary care (R03, Chen and Williams)	Primary care	Prolonged exposure; cognitive processing therapy	Veterans Affairs SDM^j^ protocol	Discover: HCD approach interviews with 22 clinicians and 25 patients with PTSD Design/build: usability testing of iterative adaptations of an electronic health record template for conducting SDM with primary care–based mental health clinicians	12
Modification of a parenting intervention for primary care–based delivery to women with perinatal depression and anxiety: PFR^k^ (R03, Bhat and Oxford)	Primary care and prenatal clinics	PFR	N/A	Discover: focus group with users of PFR to identify PFR features to be modified Design/build: iterative design of the PFR-Brief protocol in collaboration with an end user participatory design group alternating with consumer feedback in microtrials	3
Supporting iterative design of homework in problem solving therapy (R03, Agapie and Areán)	Individual therapy sessions with older adults in an urban setting	PST	N/A	Discover: interviews with patients and analysis of recordings of sessions [52].	20
Improving usability of a comprehensive self-management intervention to address anxiety and depression among persons with irritable bowel syndrome (R03, Kamp and Levy)	Primary care and gastroenterology clinics across the Pacific Northwest	Comprehensive self-management intervention for irritable bowel syndrome	N/A	Discover: interviews with 12 patients and 14 health care providers Design/build: usability testing of an interactive digital prototype	5
Iterative (re)design of a virtual postpartum depression intervention with Latina mothers (R03, Gonzalez and Ramirez)	Digital space (Ginger.io)	Mothers and Babies Program	N/A	Discover: surveys and qualitative interviews with Latina mothers in the postpartum period Design: prototype of a web-based mental health platform	4

^a^EBPI: evidence-based psychosocial intervention.

^b^BA: behavioral activation.

^c^PST: problem-solving therapy.

^d^N/A: not applicable.

^e^mHealth: mobile health.

^f^RUBI: Research Units in Behavioral Intervention.

^g^CAMS: Collaborative Assessment and Management of Suicidality.

^h^SSF: Suicide Status Form.

^i^PTSD: posttraumatic stress disorder.

^j^SDM: shared decision-making.

^k^PFR: Promoting First Relationships.

A total of 6 projects also recommended redesigns based on technology. This reflects a few factors internal and external to the Center. Internally, researchers from the Paul G Allen School of Computer Science and Engineering and the Department of Human Centered Design and Engineering—2 units extensively focused on the study and design of sociotechnical systems—were central to our team. Externally, technology is often an appealing way of increasing the reach of interventions (although, as discussed in the *Results* section, this can sometimes be too optimistic a view [53]). This became even more important as many projects were operational during the COVID-19 pandemic, making telehealth and remote care more common.

Finally, the overarching Center mission—the identification of usability challenges in underresourced, non–mental health settings—informed the selection of the projects included in this study. Teams were led by investigators from different disciplines, many of whom were from HCD and technology design fields; as a result, solutions identified may have been informed by that discipline. Investigators proposed projects with the intent of uncovering usability challenges where they saw opportunities for improvement and redesign of interventions or implementation strategies rather than focusing on the identification of intervention or strategy features that were usable. The Center’s focus on nonspecialty settings also influenced which issues were identified: many of the EBPIs and implementation strategies studied may work in specialty mental health settings but become unusable in the nonspecialty settings where most people access care.

### Adaptation of Usability Issue Concept and Reporting Format

We adapted a common usability issue–reporting format drawn from HCD and human-computer interaction. This template was adapted by the center’s methods and design team and solicited the following information:

- Descriptive title of the issue or problem
- Severity of the problem, rated from catastrophic to subtle on a scale adapted from a study by Dumas and Reddish [54]; this resulted in a 5-point scale, where L0=catastrophic, risks causing harm; L1=prevents completion of task; L2=causes significant delay and/or frustration; L3=minor effect on usability; and L4=subtle, possible future improvement
- Scope of the problem: who is affected (all users or some) and how much (eg, every session or just when starting and a particular module in an intervention or widespread)
- Complexity of the issue: how straightforward it would be to address the issue, which includes both how well-understood the problem is and how much it interacts with different components of the EBPI or implementation strategy
- Evidence for the issue: includes both qualitative and quantitative evidence (as available) to support the reader in understanding the problem, who it affects, and its consequences

If the teams knew of related research, we encouraged them to reference it. We also encouraged them to describe any next steps or known solutions. Our initial format drew on SM’s experience in teaching usability and EF’s expertise as a user experience professional.

### Development of the Usability Issue Format

We piloted the usability issue format with 2 of the smaller-scale projects, asking investigators to report issues from their data using a survey and accompanying guidance on usability issue reporting. We then examined these preliminary issues to clarify guidance and added examples in a survey that we would use with all teams. Changes made at this stage included describing problems with the intervention or implementation strategy rather than with users and revising to be as specific as possible in describing the affected components of the intervention and/or implementation strategy and the consequences. This also led us to suggest describing problems in the following format: *When [PRECURSOR], the [COMPONENT] is / has / is experienced as / results in / etc. [PROBLEM] which [CONSEQUENCE].* Multimedia Appendix 1 [33,54,55] presents the final usability issue survey and guidance.

### Usability Issue Identification and Reporting

We introduced the usability issue survey and guidance at a workshop for all Center investigators. During the workshop, we presented example issues and revisions that would make them more precise and attribute faults to the EBPI or implementation strategy rather than to interventionists or recipients. We encouraged investigators to apply the survey to any preliminary results they had and to ask questions.

Following the workshop, we asked the investigators to use the survey to report the issues they identified in their projects. We encouraged them to complete the survey primarily after the Discover phase—the phase during which they worked to understand the current EBPI or implementation strategy’s use in its destination context—and to complete it again with any additional issues identified in the iterative design and build phases. Teams emailed the completed surveys to the Center investigators.

As individual project teams analyzed data from their projects to complete the survey, some were asked whether to report an issue according to our guidance as follows:

As a center, we are interested in usability issues with the interventions or implementation strategies [e.g., Problem Solving Therapy is fatiguing to do all day], not more run of the mill usability issues with the way it is delivered [e.g., the button should be bigger; the handout’s colors clash] unless these issues significantly interfere with a user’s ability to accomplish the core tasks of the intervention.

The criteria for issues of interest were difficult to discern in some projects. For example, when a worksheet used during an intervention did not provide enough space for recipients to respond to a question, Center and project researchers determined that this should not be reported through consensus. However, when the same worksheet consistently did not support the key elements of an intervention, the team determined that this was an important finding to report.

For many investigators, this project was their first exposure to the concept of usability and reporting on usability issues; therefore, we found it necessary for Center researchers (AL, SM, and EF) to work iteratively and collaboratively with teams to refine usability issues before finalizing them. Common challenges included framing usability issues in a way that blamed the interventionist or recipient (eg, their lack of training or time rather than the EBPI or strategy requiring more training or timing than someone had), writing compound usability issues that could be separated, and describing the potential solution rather than the issue that necessitated a solution.

### Analysis of Usability Issues From Projects: Affinity Diagramming Process

Using an affinity diagramming process [56], we coded the usability issues reported by each project to identify clusters of usability issues. We recorded 72 issues from 11 projects as sticky notes in a digital, collaborative space. We then created 3 copies, which 3 researchers (EF, RA, and KO) each used to independently group issues across projects and label the resulting groups. Throughout this process, researchers worked to identify themes that inhibited the usability of EBPIs and implementation strategies rather than bucketing issues according to specific parts of therapy, implementation, or particular artifacts.

The coding process comprised 3 rounds of review, starting with the initial coding and discussion among the coders (ECF, RA, and KO) to compare preliminary categories and build a consensus [57]. This resulted in 10 preliminary categories and corresponding definitions. The broader research team met and discussed the identified codes and categories. In a few instances, this meant returning to project teams to request additional clarification about the usability issue. ECF, RA, and KO incorporated this feedback in the second round of coding while still on their individual boards. In this round, coders also considered subcategories and any potentially missing high-level categories, resulting in a revised list of 12 categories.

The coders then individually recategorized usability issues into these 12 categories. Disagreements in the resulting categorization were resolved through discussion by coders, resulting in consensus coding, with a few remaining disagreements that were deliberated with the broader research team. To address some issues, the coders asked for further clarifications from the project teams. Through this process, we came to see 7 reported issues as *compound issues*; that is, they were multiple issues, and thus, we broke them apart. As we completed this coding, 2 project teams reported an additional 11 issues that fit within the existing categories. The final list included 90 issues.

After reviewing the final codes, the researchers suggested that each category of issues might reflect one or more design heuristics. On the basis of this discussion, the coders examined whether and how these categories were mapped to 3 sets of heuristics: the classic 10 usability heuristics by Nielsen [33], the 15 principles for good service design [58], and initial heuristics for implementable EBPIs [13]. The research team developed draft heuristics based on the sets and categories that did not seem to be covered by existing heuristics. The research team continued to refine the heuristics as we developed this manuscript.

### Ethics Approval

Individual projects were reviewed or determined exempt by the University of Washington (study numbers 2824, 3795, 4236, 4274, 6044, 6748, 6839, 7463, 7735, 7754, 7853, and 9463) and Seattle Children’s (study number 1890). Each project’s protocol documents included details about sharing data with the Center for secondary analysis.

## Results

### Overview

We identified 12 categories of usability issues in the EBPIs and implementation strategies, as summarized in Textbox 1.

**Our analysis identified 12 categories of usability issues.**

Complex and/or cognitively overwhelming: The intervention or implementation strategy is too overwhelming to the user or the interventionist.

Required time exceeds the available time: The intervention or implementation strategy demands more time than is available.

Incompatibility with interventionist preference or practice: The intervention or implementation strategy is not compatible with how the interventionist prefers—or has been trained—to work and deliver interventions.

Incompatibility with existing workflow: The intervention or implementation strategy is not compatible with the interventionists’ existing workflows.

Insufficient customization to clients or recipients: The intervention or implementation strategy cannot be tailored to client/recipient needs or does not provide enough guidance for interventionists and clients/recipients to customize it.

Intervention buy-in (value): Intervention or implementation strategy does not sufficiently build client/recipient buy-in for its value.

Interventionist buy-in (trust): The intervention or implementation strategy does not build the client’s/recipient’s trust in the interventionist.

Overreliance on technology: Intervention or implementation strategy relies on technology that creates barriers for some clinicians or recipients or that is not available to all clients or recipients.

Requires unavailable infrastructure: Intervention or implementation strategy requires physical, systemic, or organizational infrastructures that are not available.

Inadequate scaffolding for client/recipient: This involves a lack of preparation and support for the client/recipient. The intervention or implementation strategy lacks support for the client/recipient to understand and succeed in the required activities of the intervention.

Inadequate training and scaffolding for interventionists: The intervention or implementation strategy’s training and scaffolding do not provide enough initial and/or ongoing support to deliver the invention as designed or to know how to respond to emergent challenges.

Lack of support for necessary communication: The intervention or implementation strategy requires but does not sufficiently facilitate communication between interventionist and client/recipient.

### Complex or Cognitively Overwhelming

Too complex or cognitively overwhelming EBPIs or implementation strategies were the most common types of usability issues uncovered in all the projects surveyed, accounting for 12 of 90 issues submitted to the Center. In these issues, the complexity of the intervention or implementation strategy exceeded the interventionist and/or client’s ability to manage it, creating an excessive cognitive or emotional burden.

An example of this came up in PST, where therapists and clients worked together to complete a worksheet that began by asking them to clearly identify the problem the client hoped to address that week and then their goal. If the problem identification step resulted in problems that were too complex, clients and therapists could be unable to complete the next steps. Complex problems could take more time to discuss than is afforded in a session, leading to running out of time. The selection of a complex problem could also overwhelm clients, who then become stuck in the next steps, with interventionists unsure of how to best support them.

In another project that focused on a digital decision support tool to support clients with shared decision-making (SDM) and to support the initiation of and adherence to EBPIs for posttraumatic stress disorder within primary care, clinicians and clients found the presented information to be overwhelming. They reported that the tool contained too much information, too many words, charts that were too busy, and insufficient visual or audio support for interpreting this information.

### Required Time Exceeds Available Time

Of the 90 issues, 11 focused on EBPIs or implementation strategies that exceeded the time available for their delivery. Although *time* is the most commonly identified implementation barrier in the literature [26], *time* here refers specifically to excessive time demands of the intervention or implementation strategy.

This category of issues frequently occurs in projects adapting an intervention to a new setting, such as primary care, where the providers have less time with the recipient than what was originally designed. For example, although a comprehensive self-management intervention addressing anxiety, depression, and gastrointestinal symptoms among individuals with irritable bowel syndrome had previously been successful when delivered by clinicians in a research setting, delivery typically took longer than the time allotted for it in a primary care visit (<10 minutes). As a result, primary care clinicians skipped or rushed the content or abandoned the intervention altogether.

Interventions also asked too much of the interventionists or recipients between sessions. For example, one project adapted the Research Units in Behavioral Intervention program, an evidence-based parent-mediated intervention that improves disruptive behavior in children with autism, for use by educators within schools [50]. However, individualized visual support, which is a core component of the Research Units in Behavioral Intervention program, requires more time to develop than educators could allocate. Generating the content required creating the pictures, developing the visual in a computer program, printing and laminating it, cutting, and velcroing to finalize the visual. Teachers do not have the time to create these materials alongside their other responsibilities, reducing their likelihood of using this core component of the intervention.

### Insufficient Customization to Clients

The reported issues also highlighted the need for interventions to be adaptable and accessible to different client profiles (eg, age, race, learning styles, and education levels) and provide interventionists with guidance for tailoring to individual preferences and needs. The lack of customization was an issue in a project examining Collaborative Assessment and Management of Suicide, a suicide-specific intervention that enables clinicians to quickly assess and treat suicidality. This intervention is extremely trainable and resource efficient, making it appealing for use in high schools. Although initially designed for use with adults, the Collaborative Assessment and Management of Suicide sometimes used words/phrases that were too difficult or vague for high school students (eg, anguish, agitation, and significant loss). This resulted in low comprehension, less engagement, and uncertainty regarding how to answer the questions.

A project designing and evaluating asynchronous, remote support for BA with adolescents at risk for depression also identified a need for customization. Clinicians reported personalizing the manualized BA prompts that appeared on worksheets for each teenager in their current use of BA and that building them into digital tools prevented this personalization. This inflexibility created barriers to teenagers in applying the concepts to their own lives and situations.

### Incompatibility With Interventionist Preference or Practice

Usability issues also occur when an intervention or implementation strategy is not compatible with how the interventionist prefers—or has been trained—to do things. For example, some interventionists perceive the processes for PST and engagement as rigid, repetitive, or frustrating (eg, setting the agenda weekly feels repetitive). Consequently, these interventionists felt that this repetitive work was not stimulating and sometimes adapted the therapy process to skip it, reducing intervention fidelity.

Therapists also sometimes end up leading the selection of the problems, goals, and solutions when a client faces difficulty with a step in these manualized interventions, as they believe that this is a good use of their expertise to keep the process moving. However, this is a misapplication of the therapy process; the client should lead identification of problems to work on. When the interventionist takes the lead, they may select problems that are not important to the client or cause the client to lose ownership of the process and reduce the client’s self-efficacy for that or subsequent steps.

### Incompatibility With Existing Workflows

Sometimes, an intervention or implementation strategy is not compatible with the interventionists’ current workflows. This was an issue in a project focused on encouraging collaboration and task sharing between therapists and care managers in the treatment of depression and trauma in rural primary care clinics. Care managers had a variety of tasks, such as providing health education and resources to patients; thus, they could not support therapists in providing treatment to the extent that therapists expected. This highlighted the need to clarify roles, the division of labor, and workflows, including the types of cases that care managers could take on.

In another example, a project examined the use of BA to treat depression in individuals with cancer. Social workers were responsible for providing BA therapy alongside a wide range of services, including navigational support (eg, transportation, housing, and financial support). These navigational needs are often emergent and can crowd out the BA agenda for a session, preventing its successful delivery.

### Intervention Buy-in (Value)

In some situations, the value of the intervention is not clear or acceptable to the patient. In a project evaluating the usability of an existing Veterans Affairs (VA) web-based SDM aid, study participants reported that sections of the web-based decision tool contained confusing and misleading content, which undermined their interest in engaging with the support it could offer. At other times, the mechanism of an intervention can prevents buy-in. For example, some PST recipients, expecting something more similar to talk therapy, felt that the therapy was childish—or not even a therapy—because of its manualized approach and extensive scaffolding. This reduced their willingness to engage in the homework required by the therapy or even to return to subsequent sessions.

### Interventionist Buy-in (Trust)

The results also highlighted the need for EBPIs to support building rapport between interventionists and recipients. Although trust is not explicitly a step in most therapy and implementation processes, it is implied. For instance, some recipients did not trust their therapists’ expertise, did not feel comfortable sharing with therapists, or doubted whether a therapist could help.

In the project evaluating the use of homework in PST and engagement, recipients were sometimes reluctant to disclose sensitive information to therapists. When this prevented them from disclosing problems or barriers they faced in addressing problems, it prevented recipients from effectively engaging in the therapy process. Lack of rapport and trust also sometimes prevented the intervention from being completed, as designed in a project evaluating the EBPI Promoting First Relationships, a parenting intervention for women with perinatal depression and anxiety. A core component of the Promoting First Relationships intervention is recording the interaction of mothers with their infants and using the recording to evaluate and provide feedback about their interactions. Not all mothers felt comfortable enough with their providers to record and share this interaction, which caused them to miss a core element of the intervention.

### Overreliance on Technology

Other interventions and implementation strategies relied on technologies to which the intended interventionists or recipients did not have reliable access or were not comfortable using. This was particularly a barrier in rural areas and among older patients in accessing tele–mental health or web-based tools and resources, resulting in reduced access to care and support.

### Requires Unavailable Infrastructure

Projects also found that interventions or implementation strategies required physical, systemic, or organizational infrastructure that was not available to the client, recipient, or organizational context. For example, in a project examining the use of BA to treat depression in patients being treated for cancer, treatment schedules for BA and cancer sometimes did not align. When a patient completed their cancer treatment or changed cancer treatments, which could sometimes mean changing locations, this could disrupt their relationship with their BA interventionist, as access to a cancer center’s mental health team was sometimes conditioned on the patient actively being in cancer treatment with that center. BA is not designed for transitions in providers; thus, this could end access to mental health therapy before the end of the mental health treatment plan.

Supervision was a critical element of this integrated care in the project designing for collaboration and task sharing between therapists and care managers in the treatment of depression and trauma in rural primary care clinics. However, therapists who would provide the supervision did not have training as supervisors and did not have supervision as part of their job responsibilities or time allocated in their work. The lack of dedicated time and training inhibited effective coordination between therapists and care managers.

### Inadequate Scaffolding for Client/Recipient

Some interventions and implementation strategies lacked support for the recipient to understand and succeed in the core activities of the intervention. Both projects examining PST identified issues in this category. Some of the core concepts of PST, such as distinguishing among problems, goals, and solutions, were unclear to recipients. Although skilled interventionists could support navigating these distinctions during sessions, clients who remained unclear about these concepts at the end of this short-term intervention did not feel confident in applying PST skills without ongoing support. Other PST recipients were unsure whether certain topics (eg, relationship, divorce, and intimacy issues) were appropriate for PST. Without an explicit invitation to bring that topic into therapy, this delayed or prevented them from addressing problems most relevant to their needs and also caused them to become frustrated as they worked on less meaningful problems.

These projects also highlighted the need for scaffolding actions. PST, BA, and many other EBPIs require documenting and maintaining action plans during sessions so that clients can engage with them between sessions; thus, the interventionist can refer to it in subsequent sessions. These plans are not always documented or may be documented in a format only accessible to the client. This can prevent clients from pursuing plans between sessions, thus reducing the essential component of the intervention.

### Inadequate Training and Scaffolding for Interventionists

Some interventions and implementation strategies did not provide sufficient training and scaffolding for the interventionist to deliver the therapy as designed or to respond to emergent challenges.

For example, in a project to support integrated care among therapists and care managers in the delivery of BA, not all care managers had sufficient training to deliver BA confidently and with fidelity. As therapists had limited availability to provide supervision (as described under infrastructure), these care managers did not have resources to which they could reliably turn when a situation exceeded the limits of their training. This resulted in handing off the patient to a therapist rather than improving their skills.

Similarly, the VA’s SDM tool for posttraumatic stress disorder provided much information to support SDM but limited scaffolding for the process. Consequently, clinicians without training or significant experience in SDM tended to fall back on what they knew and initiated SDM less frequently in their interactions with clients.

### Lack of Support for Necessary Communication

Interventions require effective communication between the interventionist and the recipient; however, they do not always sufficiently facilitate communication. For example, the VA’s SDM tool provided much information to interventionists and recipients but did not support collaborative review and the reaching of a shared understanding of that information. This prevented the effective use of information.

The period between the sessions also presents communication challenges. Projects focused on BA noted that patients could encounter new situations or barriers to their action plans for which they were not prepared. Without a clear communication pathway to an interventionist or other asynchronous support, clients often deferred their actions until the next session. By that time, clients had trouble recounting details of the challenging situation, further limiting their ability to fully engage in treatment. Such situations interact with the infrastructure for providing mental health care; access to interventionists between sessions is certainly not always possible, but redesigns that support documentation of the situation for later recall and collaboration could better support communication.

### Summary: Severity, Scope, and Complexity

In addition to reporting issues, project teams characterized them according to severity, scope, and complexity. Table 2 summarizes these ratings.

All reported issues were of severity that prevented the completion of a task (L1; 29 issues), which caused significant delay and/or frustration for the client or recipient (L2; 50 issues), or minor annoyances (L4; 11 issues). None of the teams reported catastrophic usability issues or issues that threatened recipient safety. Teams also did not report potential future improvements; we believe this is because they focused more on identifying current barriers to the delivery of EBPIs and implementation strategies.

**Table 2:** Severity, scope, and complexity by issue category (N=90).

Category	Number, n	Severity^a^, n			Scope, n				Complexity, n		
		L1^b^	L2^c^	L3^d^	Global	Medium	Local	High		Medium	Low
Complex and/or cognitively overwhelming	12	6	4	2	10	0	2	3		5	4
Required time exceeds available time	10	4	5	1	7	1	2	3		4	3
Incompatibility with interventionist preference or practice	7	1	6	0	2	0	5	2		4	1
Incompatibility with existing workflow	4	1	2	1	4	0	0	1		0	3
Insufficient customization to clients/recipients	9	0	8	1	6	3	0	1		5	3
Intervention buy-in (value)	8	3	5	0	4	0	4	4		1	3
Interventionist buy-in (trust)	7	1	6	0	2	0	5	0		5	2
Overreliance on technology	5	3	1	1	3	0	2	2		2	1
Requires unavailable infrastructure	10	5	4	1	7	2	1	3		4	3
Inadequate scaffolding for client/recipient	6	1	2	3	3	0	3	0		2	4
Inadequate training and scaffolding for interventionists	6	4	2	0	4	2	0	2		3	1
Lack of support for necessary communication	6	0	5	1	5	0	1	2		4	0

^a^No project teams reported L0 (catastrophic, risks causing harm) or L4 (subtle, future enhancement) usability issues.

^b^Prevents completion of task.

^c^Causes significant delay and/or frustration.

^d^Minor effect on usability.

Prevalent, high-severity categories included the issues of *complex and/or cognitively overwhelming, required time exceeds available time,* and *requires unavailable infrastructure.* These were also among the categories with the highest complexity ratings. The first 2 categories were often interrelated: when part of an EBPI was complex or overwhelming for the interventionist or recipient, this would often lead to them running out of time in a session or other interactions. Alternatively, when aspects of a session not accounted for by the EBPI left less time during a session, complex components quickly overwhelmed interventionists and recipients, who tried to make the most of the limited time. In either case, the effects were severe: steps of the intervention were skipped or never started, likely decreasing its efficacy.

*Requiring unavailable infrastructure* was prevalent in projects adapting EBPIs or implementation strategies for different settings. In many of these issues, the original EBPI or implementation strategy prescribed (or assumed) resources and infrastructure that were not available in the new setting; thus, substantial redesign would be required for successful implementation. This also relates to the fourth category with high severity—*overreliance on technology*. Some interventions or implementations required technology that was not available in new settings (eg, expecting reliable, low-latency internet connectivity in rural settings) or for some users (eg, for people to have a newer device or internet connectivity at home); this distinction is also reflected in the bimodal distribution between global scope (all recipients for a setting) or local scope (just the recipients who did not have access to the necessary technology). When the intervention depended on access to such technology with no alternative ways of completing it, it prevented some recipients or entire settings from benefiting.

Finally, the different severity ratings for *inadequate training and scaffolding for interventionists* and *inadequate training and scaffolding for the client/recipient* highlight the different effects of usability issues directly affecting the recipient versus the interventionist. Interventionists with sufficient training and scaffolding could support a client through an intervention with reasonable treatment fidelity, even if the intervention’s scaffolding for the client was insufficient. However, when the interventionist did not have sufficient training or scaffolding, they could quickly become frustrated and abandon the intervention or continue delivering it but with low fidelity, thus likely decreasing its efficacy.

The issues also varied in scope. Scope can refer to either how widespread an issue is with respect to a service or product design *or* what proportion of users are affected. Among the reported issues, the project teams weighed the user group most heavily in assessing the scope. Of the 90 issues, 57 issues were global in scope. These issues affected everyone within and across user groups (eg, the infrastructure or necessary scaffolding was not available to anyone in the project setting or something about the intervention or implementation’s design was overwhelming or too time intensive for all interventionists or all recipients in a setting). Of the 90 issues, 25 issues were local in scope. These issues affected only a specific user group (eg, were not culturally responsive for *some* recipients in a setting or required a technology that only some interventionists or recipients lacked) or one aspect of the intervention or implementation strategy with which only some interventionists or recipients interacted. The remaining 8 issues were medium in scope. These issues tended to affect all interventionists or recipients to some degree but were only severe for some.

Categories with the highest complexity included *complex and or cognitively overwhelming* and *required time exceeding available time*, as modifications to address these issues could affect the overall delivery and flow of an intervention. Some also required consideration of how much time and cognition an intervention could demand alongside other activities that might need to happen in nonspecialty care. However, even these categories included medium- and low-complexity issues that project teams believed they could address with simpler modifications to the intervention or implementation strategy. In terms of severity, issues that affected the interventionist tended to be more complex than those that affected the client; taken together, this emphasizes how a well-prepared interventionist can smooth out rough edges in an intervention for a client while usability issues for the interventionist flow through also become issues for the recipient.

The importance of designing to support the therapist has been noted in previous work on digital mental health technologies, which emphasizes the need to build on their existing workflows, avoid burdensome time demands, and support communication, although within reasonable boundaries [35]. We found that these needs are not unique to digital interventions, and usability issues related to these design principles are present in many EBPIs and implementation strategies.

## Discussion

### Principal Findings

Our results indicate widespread usability challenges for EBPIs and their implementation strategies. To some extent, this is a result of the way the Center shaped the research: investigators looked to use cases and settings in which people were known or anticipated to experience difficulties, with the goal of mitigating those difficulties and improving outcomes. However, they are consistent with other research noting usability barriers to the successful implementation and use of EBPIs [39,59,60], as well as other health interventions in complex settings [61]. Addressing these issues is an urgent concern for improving mental health care in nonspecialized settings.

Although a comprehensive discussion of how to redesign to address the identified usability issues is beyond the scope of this paper, we note that addressing these usability issues in intervention design and implementation may nonetheless not be easy. Several issues point to the need for interventions to better fit individual provider preferences and workflows, the context of a particular clinic, or the constraints of an individual patient, all of which might suggest more tailorable or customizable interventions and implementations. This parallels earlier guidance for digital mental health interventions to be more adaptable [35]. However, added customizability often drives complexity—to borrow from the discussion of customization in intelligence interfaces by Woods [62]: “[customization] is likely to provide the illusion of assistance while creating a new layer of burdens and complexities.” This challenge is particularly salient as other usability issues point to the need to reduce the complexity of interventions so that they are less overwhelming during and between sessions. Innovative intervention designs and implementation strategies may yet be able to achieve both of these goals; however, it will be more difficult than addressing only one.

In addition, although previous research has examined the role of buy-in for both adopting a new or changed intervention or implementation strategy in an organization [63,64] and for whether someone seeks a particular health intervention, our results emphasize the need for ongoing attention to buy-in to support engagement. In the projects in our study, interventionists and recipients typically expressed initially favorable reactions to an intervention or implementation strategy—interventionists were seeking better practices, and recipients, as noted in the *Introduction* section, favored psychosocial interventions over alternatives but then found their confidence in the intervention, or the interventionist’s ability to deliver it, waning as they engaged with specifics. Addressing these challenges is important to sustain engagement.

Our work with 13 project teams and the results of their work add to the growing literature on the value of adapting methods and constructs from HCD to examine and design EBPIs and implementation strategies. Our approach is consistent with the view that IS can contribute broad, multilevel frameworks that guide researchers and design and implementation teams, whereas HCD contributes specific methods to engage and learn from users [65-67]. That previous work also noted that although the two fields are complementary, efforts to align and scaffold the work of applying them would be necessary to achieve their benefits. In our work with the ALACRITY Center projects, we found that adapting usability issue analysis and reporting surveys facilitated teams in applying this lens to their work; however, it was not sufficient on its own. Teams that did not already have HCD expertise needed support from Center researchers to select HCD methods; apply effective but efficient analysis techniques; and, in most cases, iterate with Center researchers on reported usability issues. Consequently, we urge organizations planning to apply our DDBT approach to (1) develop resources that scaffold the process for design and implementation teams and (2) ensure that HCD and IS experts are available to support the design and implementation teams in navigating key decisions if they do not have that expertise internally. We are working on this within the UW ALACRITY Center, and we believe that this work will require contributions from across the field.

### Limitations and Future Work

#### Overview

As described in the *Methods* section, Center experts extensively supported project teams as they developed their plans for assessing usability issues and as they worked to describe usability issues. On the basis of our interactions with teams, we observed tendencies that could prevent teams from working more independently from correctly attributing problems and, thus, from addressing the right problems. The most common example of this was framing usability issues in ways that attributed the problem to the user (usually an interventionist or a recipient) rather than to the intervention’s design or implementation.

In this study, we addressed these challenges through dialog and coaching. We do not assume that the same level of support would be available to all teams working on designing and implementing interventions, and future research should continue to iterate on our process and supports for identifying and acting on usability issues so that teams with less support can successfully identify and address usability issues with fewer resources.

In addition, all data were collected from projects of the UW ALACRITY Center, led by university-based researchers, and funded through a call that required interdisciplinary teams. As noted in the *Methods* section, this may have biased our results toward EBPIs and implementation strategies with opportunities for improvement, toward EBPIs in which UW researchers have expertise, and toward technology-mediated interventions.

#### Stages of Design at which Usability Should Be Assessed and Issues Addressed

The projects in our sample engaged with interventions and implementation strategies at different stages, including examining interventionist and recipient experiences with existing interventions and implementation processes and testing prototypes of changed interventions or strategies that varied in their fidelity (level of detail, completeness, and functionality).

Owing to the limitations of prototypes, people are often unable to engage with low-fidelity prototypes in situations with high external validity; thus, there is a risk that usability issues that only emerge in real-world situations will go unobserved [68]. In addition, limited engagement with a low-fidelity prototype can make it difficult to distinguish between an initial adoption barrier and an ongoing barrier to its successful use. Therefore, it is important to assess usability issues at increasing levels of fidelity and in situations with increasing external validity. This might involve moving from *laboratory-based* testing (ie, users interacting with an intervention in circumscribed scenarios) to in vivo testing with fewer controls. However, this move is likely to require additional resources as it requires real-world intervention or implementation strategy deployment.

On the basis of our experiences, we recommend assessing EBPIs and implementation strategies for usability at all stages of their design and implementation, including initial development, refinement, pilot testing, and larger deployment. HCD has developed a mature understanding of the types of questions that should or should not be asked based on a prototype’s capabilities and fidelity [69-73]. We believe that much of this guidance can be applied to EBPI design and implementation, such as the general principles articulated previously, although developing more specific guidance should be an area for future research.

#### From Issues to Designs: Heuristics and Assessing Improvements

In the usability of digital artifacts and services, research and accumulated experiences with usability issues have led to the development of *heuristics* or guiding principles for evaluating whether products are usable [74]. In addition to the classic 10 heuristics for usability engineering by Nielsen [33], there are also specialized heuristics for different types of products, such as chatbots [75], public displays [76], and voice user interfaces [77], and services [58]. Most relevant to our work, Lyon and Koerner [13] proposed preliminary heuristics for the implementability of EBPIs.

These heuristics are commonly taught for use in a process known as heuristic evaluation [78], in which experts in user research or design use them to review a prototype or product and identify potential problems. Practitioners also develop familiarity with common sets of heuristics or those specific to the domain in which they work and use them to guide design and not only evaluate it.

Previous work in implementation science began to articulate potential heuristics for the design and implementation of EBPIs [13] and used them to evaluate EBPIs [1]. On the basis of the results of our study, which contribute to an expanded understanding of usability issues, we propose a revised and expanded set of heuristics for the design and implementation of EBPIs (Table 3).

We propose that these heuristics guide the work of those designing new or refined EBPIs, as well as groups working on implementing and adapting existing EBPIs. However, a limitation of heuristic evaluations in other domains is that having a design expert examine a product is often insufficient for observing usability issues that emerge from complex behaviors or situations; therefore, in-depth usability evaluation in both controlled settings and the field remains necessary [68,79,80]. This is particularly true for EBPIs and implementation strategies, both of which are complex health interventions [81]. Future research should test whether the intervention and implementation strategy design guided by teams using these heuristics leads to better outcomes.

In addition to assessing whether the use of heuristics leads to better outcomes, future research should identify designs that are effective in addressing the common and severe usability issues identified in this study. Many of the teams that participated in this research work on doing so for their particular settings, interventions, adaptations, and users. Examining successful solutions for commonalities may lead to transferable design and implementation approaches.

**Table 3:** The proposed heuristics that could prevent or mitigate each category of usability issues.

Usability issue category	Proposed heuristic
Complex and/or cognitively overwhelming	Low cognitive load: The intervention should be simple, with clear, concise instructions, to minimize the amount of thinking required to complete a task. Minimize tasks and steps.
Time required exceeds time available	Efficiently uses time: The intervention should be designed to be completed within the time constraints of the delivery format, with attention to (1) other activities that may need to be completed in a contact point and (2) how much clients/recipients are asked to complete between contact points.
Incompatibility with interventionist preference or practice	Responsive to existing practices: Interventions should be familiar and responsive to a variety of interventionists’ work styles. *Corollary:* interventions and implementation strategies should communicate prerequisites, with respect to provider practices, for their success.
Incompatibility with existing workflow	Responsive to existing system constraints: When possible, intervention structures should be flexible to different existing workflows. *Corollary:* Interventions and implementation strategies should communicate prerequisites, with respect to provider and setting workflows, for their success.
Insufficient customization to clients	Flexible and adaptable: Interventions and their implementation strategies should be adaptable and accessible to different client/patient profiles (eg, disability, age, culture, education, or income) and provide guidance for how to match and/or adapt to appropriate clients.
Intervention buy-in (value)	Demonstrates value: The intervention goal and process should be clear and acceptable for the needs and expectations of the client/patient, and to communicate its value.
Interventionist buy-in (trust)	Satisfaction and trust: The intervention should include space for the interventionist to establish a relationship and build rapport so the client/patient can assess trust and fit.
Overreliance on technology	Avoid technology choices that exclude: Interventions mediated by, implemented in, or otherwise relying on a technology should support users with a range of ability, comfort, and access and assess whether technology prerequisites are met and, if not, either add technology support or recommend another intervention or implementation
Requires unavailable infrastructure	Minimal infrastructure: Organizational infrastructure varies and cannot be guaranteed. Interventions should have ways to assess available infrastructure and adapt to accommodate differences or recommend alternative interventions/implementations if prerequisites for success cannot be met.
Inadequate scaffolding for the client	Learnable for recipients: The intervention/tool should include elements that support the client/patient in learning the concepts and workflow necessary for the client/patient to successfully carry out their role and activities.
Inadequate training and scaffolding for provider	Learnable for interventionists: The intervention/tool should include enough training, instructions, and in the moment support so the interventionist can successfully carry out their role and responsibilities.
Lack of support for necessary communication	Enhances communication and feedback: The intervention should include mechanisms to connect the client/patient and interventionist, allow for feedback to be shared about the process, and support adjustment of the treatment plan based on what is or is not working well.

#### Expanding to Other Interventions

As noted in the *Methods section*, our sample of projects is not representative of the entire space of EBPIs and implementation strategies. It is biased by the local expertise in our Center, and consequently, EBPIs such as BA and PST are overrepresented. In addition, our Center focused on nonspecialty settings, especially primary care. Finally, most HCD experts affiliated with the Center also work in human-computer interaction; thus, of the 13 projects, 8 were oriented toward those in which investigators hypothesized that some use of technology could better support the intervention or its implementation or was already being used in this way.

We believe that the issues we describe in this paper, as well as the approaches to identifying them in other EBPIs and implementation strategies, will be found in other types of therapies, whether they use technology to support their delivery. However, future research should assess this issue. A broader understanding of usability issues in mental health care will help to better understand the prevalence, severity, and implications of different types of usability issues.

EBPIs, implementation strategies, and digital technologies are all types of health service research products that can benefit from usability evaluation [30]. Although most of our Center’s projects focused on the redesign of EBPIs by incorporating digital solutions, projects that focused directly on strategies indicate that the usability evaluation methods apply to them as well. However, some differences between EBPIs and implementation strategies may lend themselves to somewhat different testing techniques. Given that strategies relative to client-facing psychosocial interventions tend to involve a more diverse array of system levels, interested parties, and interactions, the direct evaluation of components via techniques such as behavioral rehearsals may be less feasible. Instead, implementation strategies might be most readily evaluated using techniques such as cognitive walk-through [25], which can focus on broader processes such as ways that organizational leaders influence the implementation climate.

We also anticipate that an important next step in the usability evaluation of complex health interventions such as EBPIs and strategies will be to extend this study to other domains of health. Although mental and behavioral health interventions are among the most complex in contemporary health care, often representing reciprocal, socially mediated processes delivered over many months (eg, psychotherapy protocols lasting 12-16 sessions), other fields also use interventions with a high degree of complexity (eg, 6-month lifestyle interventions for women at high risk of breast cancer [82]). Although additional research is needed to determine the extent to which these interventions might demonstrate comparable usability issues, as well as be improved with similar heuristics, it is likely that many of our findings apply to improving the intervention implementability more broadly. Some usability issue categories identified in our research (eg, overreliance on technology and unavailable infrastructure, incompatibility with interventionist preference or practice, incompatibility with a setting’s workflows, insufficient support for communication, and supporting) parallel concerns noted in other health systems research (eg, need to attend to workflows and communication; organizational policies, procedures, and culture; and computing infrastructure [61]).

### Conclusions

Previous research has indicated that usability may explain the low adoption of EBPIs in nonspecialty settings [39]. A total of 13 projects examining EBPIs and associated implementation strategies identified 90 usability issues, which our team clustered into 12 categories. Of the 90 issues, 29 could prevent the completion of part of an EBPI, and 50 could cause significant delay or frustration in care. We contribute to an approach for analyzing and reporting usability issues in future projects, categories of usability issues that EBPI and implementation strategy designers should seek to avoid, and heuristics to support more usable EBPI and implementation strategy designs.

## Appendix

Multimedia Appendix 1 Usability issue guidance and survey.

## Abbreviations

ALACRITY: Advanced Laboratories for Accelerating the Reach and Impact of Treatments for Youth and Adults with Mental Illness
BA: behavioral activation
CAMS: Collaborative Assessment and Management of Suicidality
DDBT: Discover, Design/Build, and Test
EBPI: evidence-based psychosocial intervention
HCD: human-centered design
IS: Implementation Science
PST: problem-solving therapy
SDM: shared decision-making
UW: University of Washington
VA: Veterans Affairs

## References

[1] Raue PJ, Weinberger MI, Sirey JA, Meyers BS, Bruce ML (2011). Preferences for depression treatment among elderly home health care patients. Psychiatr Serv.

[2] Arean PA, Raue PJ, Sirey JA, Snowden M (2012). Implementing evidence-based psychotherapies in settings serving older adults: challenges and solutions. Psychiatr Serv.

[3] Houle J, Villaggi B, Beaulieu M, Lespérance F, Rondeau G, Lambert J (2013). Treatment preferences in patients with first episode depression. J Affect Disord.

[4] McHugh RK, Whitton SW, Peckham AD, Welge JA, Otto MW (2013). Patient preference for psychological vs pharmacologic treatment of psychiatric disorders. J Clin Psychiatry.

[5] Quiñones AR, Thielke SM, Beaver KA, Trivedi RB, Williams EC, Fan VS (2014). Racial and ethnic differences in receipt of antidepressants and psychotherapy by veterans with chronic depression. Psychiatr Serv.

[6] Olfson M, Kroenke K, Wang S, Blanco C (2014). Trends in office-based mental health care provided by psychiatrists and primary care physicians. J Clin Psychiatry.

[7] Rones M, Hoagwood K (2000). School-based mental health services: a research review. Clin Child Fam Psychol Rev.

[8] Owens J, Lyon A, Brandt N, Warner C, Nadeem E, Spiel C, Wagner M (2014). Implementation science in school mental health: key constructs in a developing research agenda. School Ment Health.

[9] Duong MT, Bruns EJ, Lee K, Cox S, Coifman J, Mayworm A, Lyon AR (2021). Rates of mental health service utilization by children and adolescents in schools and other common service settings: a systematic review and meta-analysis. Adm Policy Ment Health.

[10] Lyon A, Ludwig K, Romano E, Koltracht J, Vander Stoep A, McCauley E (2014). Using modular psychotherapy in school mental health: provider perspectives on intervention-setting fit. J Clin Child Adolesc Psychol.

[11] Stewart R, Chambless D, Stirman (2018). Decision making and the use of evidence based practice: is the three-legged stool balanced?. Pract Innov (Washington, DC).

[12] (2015). Psychosocial Interventions for Mental and Substance Use Disorders: A Framework for Establishing Evidence-based Standards.

[13] Lyon AR, Koerner K (2016). User-centered design for psychosocial intervention development and implementation. Clin Psychol (New York).

[14] Proctor EK, Powell BJ, McMillen JC (2013). Implementation strategies: recommendations for specifying and reporting. Implement Sci.

[15] Lewis CC, Klasnja P, Powell BJ, Lyon AR, Tuzzio L, Jones S, Walsh-Bailey C, Weiner B (2018). From classification to causality: advancing understanding of mechanisms of change in implementation science. Front Public Health.

[16] Lyon AR, Munson SA, Renn BN, Atkins DC, Pullmann MD, Friedman E, Areán PA (2019). Use of human-centered design to improve implementation of evidence-based psychotherapies in low-resource communities: protocol for studies applying a framework to assess usability. JMIR Res Protoc.

[17] ISO 9241-420:2011 - Ergonomics of human-system interaction — Part 420: selection of physical input devices. ISO.

[18] Novins DK, Green AE, Legha RK, Aarons GA (2013). Dissemination and implementation of evidence-based practices for child and adolescent mental health: a systematic review. J Am Acad Child Adolesc Psychiatry.

[19] Stewart RE, Chambless DL (2009). Cognitive-behavioral therapy for adult anxiety disorders in clinical practice: a meta-analysis of effectiveness studies. J Consult Clin Psychol.

[20] Becker EM, Smith AM, Jensen-Doss A (2013). Who's using treatment manuals? A national survey of practicing therapists. Behav Res Ther.

[21] Garland A, Hawley K, Brookman-frazee L, Hurlburt M (2008). Identifying common elements of evidence-based psychosocial treatments for children's disruptive behavior problems. J Am Academy Child Adolescent Psychiatry.

[22] Shelton RC, Cooper BR, Stirman SW (2018). The sustainability of evidence-based interventions and practices in public health and health care. Annu Rev Public Health.

[23] Damschroder LJ, Aron DC, Keith RE, Kirsh SR, Alexander JA, Lowery JC (2009). Fostering implementation of health services research findings into practice: a consolidated framework for advancing implementation science. Implement Sci.

[24] Curran GM, Bauer M, Mittman B, Pyne JM, Stetler C (2012). Effectiveness-implementation hybrid designs: combining elements of clinical effectiveness and implementation research to enhance public health impact. Med Care.

[25] Lyon AR, Coifman J, Cook H, McRee E, Liu FF, Ludwig K, Dorsey S, Koerner K, Munson SA, McCauley E (2021). The Cognitive Walkthrough for Implementation Strategies (CWIS): a pragmatic method for assessing implementation strategy usability. Implement Sci Commun.

[26] Cook CR, Lyon AR, Locke J, Waltz T, Powell BJ (2019). Adapting a compilation of implementation strategies to advance school-based implementation research and practice. Prev Sci.

[27] Powell BJ, Waltz TJ, Chinman MJ, Damschroder LJ, Smith JL, Matthieu MM, Proctor EK, Kirchner JE (2015). A refined compilation of implementation strategies: results from the Expert Recommendations for Implementing Change (ERIC) project. Implement Sci.

[28] Morris ZS, Wooding S, Grant J (2011). The answer is 17 years, what is the question: understanding time lags in translational research. J R Soc Med.

[29] Cabassa L (2016). Implementation science: why it matters for the future of social work. J Soc Work Educ.

[30] Lyon AR, Dopp AR, Brewer SK, Kientz JA, Munson SA (2020). Designing the future of children's mental health services. Adm Policy Ment Health.

[31] Lyon A, Comtois K, Kerns S, Landes S, Lewis C (2020). Closing the science–practice gap in implementation before it widens. Implementation Science 3.0.

[32] Giacomin J (2015). What is human centred design?. Design J.

[33] Nielsen J (1994). Usability Engineering.

[34] Rieman J, Franzke M, Redmiles D (1995). Usability evaluation with the cognitive walkthrough. Proceedings of the Conference Companion on Human Factors in Computing Systems.

[35] Doherty G, Coyle D, Matthews M (2010). Design and evaluation guidelines for mental health technologies. Interact Comput.

[36] Beidas R, Cross W, Dorsey S (2014). Show me, don't tell me: behavioral rehearsal as a training and analogue fidelity tool. Cogn Behav Pract.

[37] MacLeod H, Jelen B, Prabhakar A, Oehlberg L, Siek K, Connelly K (2017). A guide to using Asynchronous Remote Communities (ARC) for researching distributed populations. EAI Endorsed Transact Pervasive Health Technol.

[38] Lyles CR, Sarkar U, Osborn CY (2014). Getting a technology-based diabetes intervention ready for prime time: a review of usability testing studies. Curr Diab Rep.

[39] Lyon A, Pullmann M, Jacobson J, Osterhage K, Al Achkar M, Renn B, Munson S, Areán P (2021). Assessing the usability of complex psychosocial interventions: The Intervention Usability Scale. Implement Res Pract.

[40] Lewis J, Sauro J (2009). The factor structure of the system usability scale. Human Centered Design.

[41] Suh H, Shahriaree N, Hekler E, Kientz J (2016). Developing and validating the user burden scale: a tool for assessing user burden in computing systems. Proceedings of the 2016 CHI Conference on Human Factors in Computing Systems.

[42] Weiner BJ, Lewis CC, Stanick C, Powell BJ, Dorsey CN, Clary AS, Boynton MH, Halko H (2017). Psychometric assessment of three newly developed implementation outcome measures. Implement Sci.

[43] Lewinsohn PM (1974). A behavioral approach to depression. The Psychology of Depression: Contemporary Theory and Research.

[44] Jacobson NS, Dobson KS, Truax PA, Addis ME, Koerner K, Gollan JK, Gortner E, Prince SE (1996). A component analysis of cognitive-behavioral treatment for depression. J Consulting Clin Psychol.

[45] Jacobson NS, Martell CR, Dimidjian S (2006). Behavioral activation treatment for depression: Returning to contextual roots. Clinical Psychology: Science and Practice.

[46] D’Zurilla TJ, Nezu AM (2010). Problem-solving therapy. Handbook of Cognitive-Behavioral Therapies.

[47] Bhattacharya A, Nagar R, Jenness J, Munson S, Kientz J (2021). Designing asynchronous remote support for behavioral activation in teenagers with depression: formative study. JMIR Form Res.

[48] Jenness JL, Bhattacharya A, Kientz JA, Munson SA, Nagar R (2022). Lessons learned from designing an asynchronous remote community approach for behavioral activation intervention for teens. Behav Res Ther.

[49] Ben-Zeev D, Meller S, Snyder J, Attah D, Albright L, Le H, Asafo S, Collins P, Ofori-Atta A (2021). A digital toolkit (m-healer) to improve care and reduce human rights abuses against people with mental illness in West Africa: user-centered design, development, and usability study. JMIR Ment Health.

[50] Bearss K, Tagavi D, Lyon AR, Locke J (2022). Iterative redesign of a caregiver-mediated intervention for use in educational settings. Autism.

[51] Increasing the usability and cultural responsiveness of a suicide-specific treatment for high schools. School Mental Health Assessment Research & Training Center.

[52] Agapie E, Areán P, Hsieh G, Munson S (2022). Longitudinal goal setting: a holistic, longitudinal process of transformative understanding in mental health. Proceedings of the 25th ACM Conference On Computer- Supported Cooperative Work And Social Computing.

[53] Graham AK, Lattie EG, Powell BJ, Lyon AR, Smith JD, Schueller SM, Stadnick NA, Brown CH, Mohr DC (2020). Implementation strategies for digital mental health interventions in health care settings. Am Psychol.

[54] Dumas J, Redish J (1999). A Practical Guide to Usability Testing.

[55] Lavery D, Cockton G, Atkinson M (1997). Comparison of evaluation methods using structured usability problem reports. Behav Inf Technol.

[56] Holtzblatt K, Wendell JB, Wood S (2004). Rapid Contextual Design: A How-to Guide to Key Techniques for User-Centered Design.

[57] Hill C, Knox S, Thompson B, Williams E, Hess S, Ladany N (2005). Consensual qualitative research: an update. J Counsel Psychol.

[58] Downe L (2020). Good Services.

[59] Lyon A, Koerner K, Chung J (2020). Usability Evaluation for Evidence-Based Psychosocial Interventions (USE-EBPI): a methodology for assessing complex intervention implementability. Implement Res Pract.

[60] Eisman A, Kilbourne A, Greene D, Walton M, Cunningham R (2020). The user-program interaction: how teacher experience shapes the relationship between intervention packaging and fidelity to a state-adopted health curriculum. Prev Sci.

[61] Sittig DF, Singh H (2015). A new socio-technical model for studying health information technology in complex adaptive healthcare systems. Cognitive Informatics for Biomedicine: Human Computer Interaction in Healthcare.

[62] Woods DD (1993). The price of flexibility. Proceedings of the 1st international conference on intelligent user interfaces.

[63] Or C, Dohan M, Tan J (2014). Understanding critical barriers to implementing a clinical information system in a nursing home through the lens of a socio-technical perspective. J Med Syst.

[64] Holden RJ, Or CK, Alper SJ, Joy Rivera A, Karsh B (2008). A change management framework for macroergonomic field research. Appl Ergon.

[65] Chen E, Neta G, Roberts M (2021). Complementary approaches to problem solving in healthcare and public health: implementation science and human-centered design. Transl Behav Med.

[66] Dopp AR, Parisi KE, Munson SA, Lyon AR (2019). A glossary of user-centered design strategies for implementation experts. Transl Behav Med.

[67] Dopp AR, Parisi KE, Munson SA, Lyon AR (2020). Aligning implementation and user-centered design strategies to enhance the impact of health services: results from a concept mapping study. Implement Sci Commun.

[68] Goodman E, Kuniavsky M (2012). Observing the User Experience: a Practitioner's Guide to User Research.

[69] Virzi R (2016). What can you learn from a low-fidelity prototype?. Proc Human Factors Society Annual Meeting.

[70] Rudd J, Stern K, Isensee S (1996). Low vs high-fidelity prototyping debate. Interactions.

[71] Catani M, Biers D (2016). Usability evaluation and prototype fidelity: users and usability professionals. Proc Human Factors Ergonomics Society Annual Meeting.

[72] Houde S, Hill C (1997). What do prototypes prototype?. Handbook of Human-Computer Interaction (2nd Edition).

[73] Sauer J, Seibel K, Rüttinger B (2010). The influence of user expertise and prototype fidelity in usability tests. Appl Ergon.

[74] Nielsen J, Molich R (1990). Heuristic evaluation of user interfaces. Proceedings of the SIGCHI Conference on Human Factors in Computing Systems.

[75] Sanchez-Adame L, Mendoza S, Urquiza J, Rodriguez J, Meneses-Viveros A (2021). Towards a set of heuristics for evaluating chatbots. IEEE Latin Am Trans.

[76] Somervell JP, Wahid S, McCrickard DS (2003). Usability Heuristics for Large Screen Information Exhibits. Proceedings of the International Conference on Human-Computer Interaction.

[77] Maguire M (2019). Development of a heuristic evaluation tool for voice user interfaces. Design, User Experience, and Usability.

[78] Nielsen J (1994). Heuristic evaluation. Usability Inspection Methods.

[79] Jordan PW, McClelland IL, Thomas B, Weerdmeeste BA (1996). Usability Evaluation In Industry.

[80] Duh HB, Tan GC, Chen VH (2006). Usability evaluation for mobile device: a comparison of laboratory and field tests. Proceedings of the 8th conference on Human-computer interaction with mobile devices and services.

[81] Litaker D, Tomolo A, Liberatore V, Stange K, Aron D (2006). Using complexity theory to build interventions that improve health care delivery in primary care. J Gen Intern Med.

[82] Han CJ, Korde LA, Reding S, Allott K, Van Doren M, Schwarz Y, Vaughan C, Reding KW (2018). Investigation of a lifestyle intervention in women at high risk of breast cancer. West J Nurs Res.

