# Supplementary material for: Usability Issues in Evidence-Based Psychosocial Interventions and Implementation Strategies: Cross-project Analysis
Source: J Med Internet Res. 2022 Jun 14;24(6):e37585. doi: 10.2196/37585 (PMC9240934; doi:10.2196/37585)
Supplement: Multimedia Appendix 1 [file jmir_v24i6e37585_app1.pdf]

The Anatomy of a Usability issue

Goal

Across the ALACRITY Center, we want to identify recurring usability issues with evidence-based practices and the services and systems designed to support or implement them, and to identify effective patterns for addressing those issues.

Many usability studies seek to improve the next iteration of the design of a service, system, or artifact. Within individual R03 and R34 projects, each team will identify and work to review their own usability issues. As a result, we would like each team to adopt a semi-standardized way of reporting usability issues to the UWAC methods core. We hope this format will also be useful for each internal team documentation and not a duplication of effort.

The UWAC has adopted the following definition of usability issues: *Aspects of the intervention and/or a demand on the user which make it unpleasant, inefficient, onerous, or impossible for the user to achieve their goals in typical usage situations (Lavery, Cockton, & Atkinson, 1997).*

As a center, we are interested in usability issues with the interventions or implementation strategies (e.g., Problem Solving Therapy is fatiguing to do all day), *not* more run of the mill usability issues with the way it is delivered (e.g., the button should be bigger; the handout's colors clash) unless these issues significantly interfere with a user's ability to accomplish the core tasks of the intervention.

When should you report usability issues?

Whenever appropriate! In general, **we recommend reporting new issues at the end of the discover phase and at the end of the build phase, as relevant to your project.**

Issues reported from the discover phase are likely to be issues with existing EBPIs and mechanisms for delivering them. Issues reported from the test phase are more likely to be about your adapted intervention or implementation, but you may also find new underlying issues with the EBPI.

We anticipate that you will be more likely to *update* previously identified issues during the design and build phases (e.g., you develop a potential solution to a previously identified issue), but you may also identify new ones there.

UW ALACRITY CENTER

Specifying Usability Issues

In the UWAC, specification of usability issues includes the following elements: (1) Description, (2) Severity, (3) Scope, (4) Complexity, and (5) Evidence. These issues may also be linked to (6) known research (e.g., previous documentation in other studies). Ultimately, (7) redesign solutions should be identified to address the highest priority issues.

These elements are presented in the table below and then described in greater detail. Elements are based on common ways of reporting usability issues in human centered design of software systems, but we have customized them to better reflect the kinds of issues we will identify in ALACRITY center projects.

**Required Elements**	
**Description**	A concise summary of what's going wrong. Aim for 1-2 complete sentences, using the following structure (explained further below):<br><br>When [PRECURSOR(S)], the [COMPONENT] is / has / is experienced as / results in / etc. [PROBLEM] which [CONSEQUENCE].<br><br>Do <u>not</u> include or imply a proposed solution in the issue description.
**Severity**	For each identified usability issue, we ask that teams assign a severity rating using the following categories (adapted from Dumas & Redish, 1999):<br><br>● Level 0 - catastrophic or dangerous; causes harm; high risk<br>● Level 1 – prevents completion of a task<br>● Level 2 – creates significant delay and frustration<br>● Level 3 – has a minor effect on usability<br>● Level 4 – subtle problem, points to a future enhancement<br><br>We recommend that, when appropriate, multiple team members independently rate each issue using these categories (see below for additional guidance).
**Scope**	Usability issues can be considered on a spectrum from **local** (i.e., confined to one user group or component of an intervention/strategy) to **global** (i.e., experienced by most/all users and pervasive across components). For this section, articulate:<br><br>● Prevalence of <u>users</u> encountering the problem (and to which user groups they below) - All, Most, Some, Few, and any particular user

	group? If it's *easy* for you to add a number, that's great - otherwise categorical is useful.<br>● Which components (including content elements, structures, artifacts, and parameters) are affected?
**Complexity**	Complexity refers to how straightforward (or not) it is to address an issue.<br><br>An issue might have low complexity if you understand the root cause of the problem and solutions are known (e.g., rewording a worksheet prompt avoids a misunderstanding).<br><br>An issue may have higher complexity if the root cause is not understood (i.e., more research is needed), if addressing the issue is likely to likely to cause other, downstream problems (i.e., there are interaction effects between the component of the intervention with the issue and other components or the health system as a whole), or if the solution is not well understood.<br><br>We recommend writing this qualitatively, e.g., "This issue has [low/medium/high] complexity, because…". Often the *because* is more important than the actual rating.
**Evidence**	Describe the qualitative and/or quantitative data that provided evidence for the usability issue and which support - and provide further understanding of - the description, severity, scope, and complexity indicated above. If possible, specify the following…<br><br>● Whether the usability issue was independently *observed* (e.g., during user interactions with a prototype) versus *reported* by a user.<br>● Whether the usability issue was *experienced* by a user versus *anticipated* based on a hypothetical situation.
Optional Elements	
**Related research**	If you have seen this kind of issue before in related research, including theoretical frameworks or models that could help us understand what's going on, we'd appreciate a citation! Similarly, if this is an example of a common heuristic, e.g., Nielsen's 10, please make that connection.<br><br>If you don't see connections, don't worry -- that's part of our job in the center core.<br><br>(Wherever you are on this)
**Recommended next steps**<br><br>**or**	If you have not verified a solution, you might recommend that one or more alternative designs be evaluated, or you might recommend that the team work on redesigns. Sometimes you have to recommend further study/usability testing to better understand the issue.

UW ALACRITY CENTER

**How your team solved the problem**	If you have tested and verified that a redesign fixes this problem, you might recommend that be implemented.<br><br>It's possible you aren't sure what the next steps are, in which case you might seek consultation from the UWAC Methods Core. Alternatively, the issue might be sufficiently minor that you don't plan to address it.

Below, we present guidance on each of these areas. Overall, the guidance aims to help researchers and intervention designers prioritize issues and understand them well so they can plan their next steps.

(1) Description

When [PRECURSOR], the [COMPONENT] is / has / is experienced as / results in / etc. [PROBLEM] which [CONSEQUENCE].

COMPONENTS of the intervention should be detailed using the same structure reflected in the DDBT intake form, including (1) *content elements* (discrete techniques), (2) *structures* (processes that guide the selection and delivery of content), (3) *artifacts* (tangible, digital, or visual materials), or (4) *parameters* (static properties that define and constrain the intervention or service "space").

Examples:

<u>Problem-solving therapy (intervention)</u>: When treating depression in community settings, clinicians experience the need to exactly follow this seven step process each time as tedious and burdensome, which results in clinician exhaustion or boredom.

<u>Post-training consultation strategy (implementation strategy)</u>
When clinicians are engaged in live consultation (*precursor*), the approach to case discussions (*component [content element]*) assumes that clinicians know how to do a concise case presentation (*problem*). When it's not concise, discussion overflows into other consultation activities (*consequence*).

When writing your description, avoid the <u>following pitfalls</u>:

- Don't start with [THE INTERVENTION]...
  - Instead, start with [COMPONENT OF THE INTERVENTION]
  - So, instead of *"Problem Solving Therapy was experienced as…."* start with *"The problem solving processes introduced in PST…"* (content element) or *"The number of sessions required for PST…"* (parameter)

- Don't focus just on one consequence if there are multiple consequences

- Different professionals will work with it in different ways, and consequences affect different stakeholders differently.

- Don't be vague in problems or consequences
  - Avoid vague language about "difficulties," "problems," etc..If you don't have the information to be more specific, more user evaluations may be needed and, should then be recommended as a next step.
  - "This takes too much time" is too vague because you don't know what to do next or what the consequences of it taking too long are. If could mean…
    - "The structure of the therapy doesn't allow enough time to get to know to my patients"
    - "Takes more sessions than I have with my patients, since they don't see me regularly"
    - "Takes longer than the session hour, so clinicians and patients don't get everything done in a session"
    - "Takes longer than the session hour, so clinicians often have to take over parts of the therapy that my clients really should be leading"

- Don't accept at face value reports from one stakeholder group about usability issues that might be experienced by another group. A stakeholder group (e.g., clinicians) often can describe experiences of other stakeholders (e.g., patients), but they are often only able to recount only an incomplete picture. You will ultimately want to test any assumptions about how a particular user group will respond by engaging in user research with that group.

Additional guidance:

- If indicated, you may reference how the information was obtained in the description of the usability issue (e.g., "clinicians reported…")
- Especially later in a redesign process, usability issues may be described in a way that is *comparative* (i.e., "X is less XXXXX than Y because XXXXXX").

(2) Severity

Often, severity is reported here. There are many severity scales, often numeric – give the label, not just the number to avoid confusion. Some teams separately report severity, complexity, and scope either as scales or descriptively.

Severity helps prioritize the fixing of problems and allocation of resources to fixing them. There are various scales, some developed more for interfaces, others that apply more broadly. Example scales include:

- 3 point: disaster, serious, cosmetic
- 5 point: catastrophic, major, medium, minor, cosmetic

We generally recommend Dumas and Redish's four-level scale, with a modification to account for how some usability issues can cause harm:

- Level 0 - catastrophic; causes harm; high risk
- Level 1 – prevents completion of a task
- Level 2 – creates significant delay and frustration
- Level 3 – has a minor effect on usability
- Level 4 – subtle problem, points to a future enhancement

It offers a reasonable level of precision, without being overwhelming. Additionally, the descriptors are broad apply not to just to screen-based interfaces.

Because there are multiple scales, we recommend reporting the full descriptor or a short name (e.g., “4 - subtle”) rather than just numbers (e.g., “4”).

We recommend that multiple team members rate each issue, if the team has sufficient expertise to do so. Methods of integrating these ratings could include:

- Calculating averages across raters
- Meeting to arrive at ratings via consensus discussions

(3) Scope

To what extent is the problem present in the product or experience? This can refer to prevalence across the system, service, or artifact as well as the prevalence of how many users / stakeholders it affects.

- **Local** – isolated to one page or section; for a particular stakeholder group
- **Global** – throughout the interface or experience; for all participants

We’d love to know number of users and from which groups, if it’s *easy* for you to add that number. If not, estimated prevalence and if issues occur in certain groups is also useful.

(4) Complexity

How difficult is the problem to understand or reproduce? How easy is it to fix? Generally, more complex problems will take more time and resources to fix, often starting with more detailed study of what is going on.

In a **low complexity problem**, it is always present and easily explicable. You know what is happening and why. You likely have an idea for how to fix it.

In a **high complexity problem**, it may be intermittent. People may be making mistakes and you don’t know exactly why or when. You may not understand how to fix it, or you may, but know that it is difficult and requires, for example, a reordering of all steps in a service. It may even require rethinking the health system.

(5) Evidence

As a minimum, we ask that you provide exemplary evidence. The goal of this evidence is to help others understand the problem, and the standard should be that it is pursuasive and informative to someone who has to redesign the intervention as a result.

Often, the most informative evidence combines data from different sources: e.g., qualitative and quantitative data from usability testing, quantitative data about the number of patients who complete versus drop out of the treatment after certain number of sessions.

As you decide which evidence to include, consider the other categories. Together, the set of evidence should illustrate the component, the issue, the consequences, their severity, and the complexity.

We do not need all *of your evidence on an issue here.* That said, if you find it easier to present all the evidence, we're happy to work with it.

(6) Related Research

Do you think this problem connects to other research? If so, we would appreciate pointers to the literature. If not, that's where our work as a center will pick up.

(7) Next Steps / Redesign

What next steps should another team working in this area do? Do you have a fix? Do you have a fix that has been validated? If so, that's great. If not, what are the next steps to creating a fix? It is common for an issue to need more study before a team is ready to redesign a service, product, or intervention.

As projects move from stage-to-stage, we anticipate that you may revisit and revise these. For example, during testing, you may confirm that a proposed solution works. Alternatively, you might identify new information that indicates the usability issue was not what your team first thought. Please keep us updated as you learn!
